# Fatal *Vibrio vulnificus* Bacteremia in Two Cirrhotic Patients with Abdominal Pain and Misty Mesentery

**DOI:** 10.5811/cpcem.2017.5.33420

**Published:** 2017-10-06

**Authors:** Ket-Cheong Lim, Yin-Ling Tan, Chin-Chu Wu, Li-Wei Lin

**Affiliations:** * Shin Kong Memorial Wu Ho-Su Hospital, Department of Emergency Medicine, Taipei, Taiwan; † Shin Kong Memorial Wu Ho-Su Hospital, Department of Medical Imaging, Taipei, Taiwan; ‡ Fu Jen Catholic University, School of Medicine, New Taipei City, Taiwan

## Abstract

Two cirrhotic patients with unexplained abdominal pain deteriorated rapidly and fatally after presenting to our emergency department. Abdominal computed tomography in both patients showed “misty mesentery”, which could not be explained by other etiologies. Both blood cultures revealed *Vibrio vulnificus*, which suggested the possible correlation of CT-finding and bacteremia.

## INTRODUCTION

*Vibrio vulnificus* infection is uncommon but potentially fatal.^1^ *V. vulnificus* is a Gram-negative, curved, rod-shaped bacterium found in warm seawater. *V. vulnificus* infections are generally acquired by eating contaminated raw seafood or through wound contamination by seawater or shellfish. These infections can result in three distinct syndromes: primary septicemia, wound infections, and gastrointestinal illness.^2^ We report two cirrhotic patients who presented with acute abdomen and “misty mesentery” on computed topography (CT), progressively worsening lactic acidosis and rapid demise in association with *V. vulnificus* bacteremia.

## CASE REPORT

### Case #1

A 50-year-old man complained of abdominal pain with radiation to the back for four hours, and one episode of blood-streaked vomitus. He presented to the emergency department (ED) at night after having consumed alcohol during the day. His medical conditions included alcoholic liver cirrhosis, alcoholic pancreatitis, peptic ulcer disease, and prior cholecystectomy.

**Image 1:**
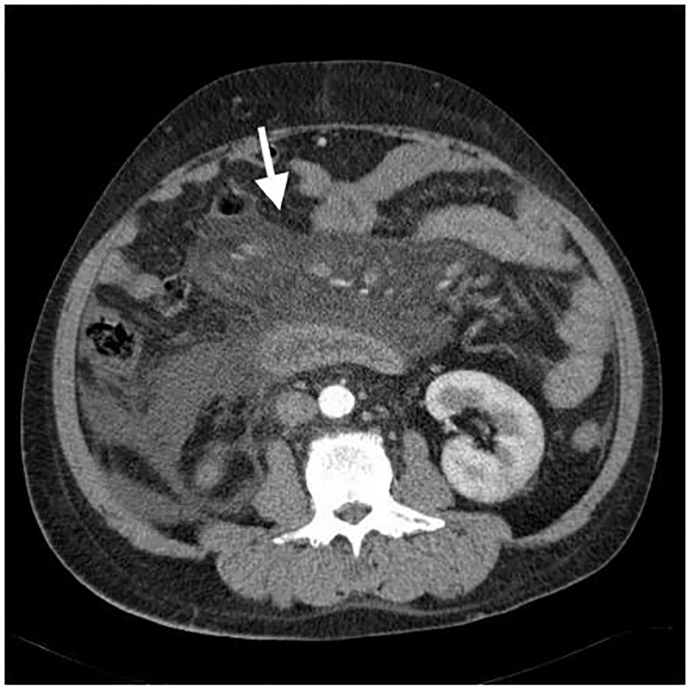
Computed tomography showing “misty mesentery” fat stranding (arrow) surrounding mesenteric vessels with ascites

Initial examination of his vital signs revealed that he was febrile (39·3°C) and tachycardic (pulse, 132 beats per minute), but without hypotension. Physical examination revealed diffuse abdominal tenderness without guarding. His hemoglobin levels were 13·2 g/dL, and his white blood count (WBC) was 3500/uL with bandemia (band: 22%). His serum lipase was normal, but hyperlactatemia (41·4 mg/dL), elevated aspartate aminotransferase (220 U/L), and hyperbilirubinemia (total bilirubin: 19·65 mg/dL) were noted. Because of intractable abdominal pain, abdominal CT was ordered, which revealed mesenteric vessels surrounded by new fat stranding with ascites when compared with his abdominal CT from four years prior (Image 1). He was subsequently admitted to the intensive care unit because of worsening hyperlactatemia (89·7 mg/dL) with newly developed metabolic acidosis (venous pH: 7·169 and bicarbonate (HCO3^-^): 14·1 mmol/L). No bullae or rash was revealed or documented during the whole process. Unfortunately, he expired nine hours after arrival, despite aggressive resuscitation and antibiotics. Blood culture analysis three days later revealed *V. vulnificus* growth*.*

### Case #2

A 61-year-old man presented to the ED with one episode of hematemesis four hours prior and subsequent epigastric abdominal pain. He had a history of alcoholic liver cirrhosis, peptic ulcer disease, and type 2 diabetes and had received radiotherapy for esophageal cancer two years prior. The patient was oriented, afebrile, and tachycardic (pulse rate, 114 beats per minute), with normotension. Laboratory studies showed bandemia (WBC: 4300/uL, band: 19%) without any reduction in hemoglobin (10·9 gm/dL, same value as one year prior), acute kidney injury (creatinine: 3·4 mg/dL), and hyperlactatemia (67·3 mg/dL). Proton-pump inhibitors and terlipressin were administered intravenously.

**Image 2:**
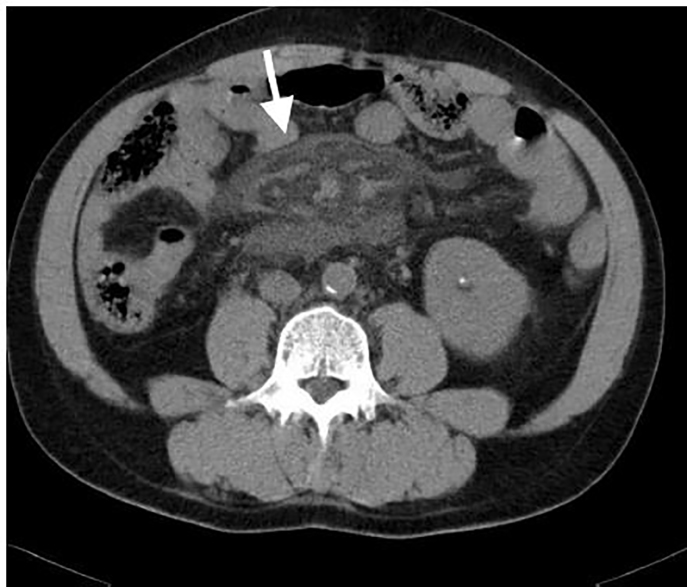
Computed tomography showing “misty mesenteric” vessels surrounded by fat stranding (arrow)

Due to intractable pain emergent CT was arranged, revealing mesenteric vessels surrounded by new fat stranding compared with CT one year prior (Image 2). An endoscopy identified angiodysplasia over the lower esophagus and duodenal ulcer without active bleeding. Eleven hours after the patient’s arrival at the ED, he became agitated and multiple areas of ecchymosis developed over his limbs. Neutropenia with worsening bandemia (WBC: 2500/uL, band: 34%), hyperlactatemia (153 mg/dL) with severe metabolic acidosis (venous pH: 7·030, HCO3^-^: 8·6 mmol/L), deteriorating kidney function (creatinine: 5·14 mg/dL), and disseminated intravascular coagulopathy ensued. Despite empirical antibiotics, continuous venovenous hemofiltration and intensive care, the patient expired 22 hours after his arrival in the ED. Blood culture analysis three days later revealed *V. vulnificus* growth.

## DISCUSSION

*V. vulnificus* infection has been categorized into three distinct syndromes: 1) primary septicemia related to the consumption of raw seafood; 2) wound infection related to necrotizing fasciitis and bacteremia; and 3) gastrointestinal illness without bacteremia.^2^ *V. vulnificus* septicemia is considerably more deadly than soft tissue infection, with mortality rates exceeding 50% and higher than 90% with septic shock.^3^,^4^

“Misty mesentery” is a radiological term used to describe an increase in mesenteric fat density without displacing the surrounding vessels in abdominopelvic CT.^5^ Mesenteric panniculitis is one of an extensive range of disorders that show misty mesentery in CT, but other possible etiologies, such as edema, hemorrhage, neoplasia, lymphedema, and inflammation, should be excluded.^6^ Reported prevalence rates range from 0.16% to 7.80%.^7^ Patients may be asymptomatic or present with non-specific chronic abdominal pain. According to our research, no report has presented an association between misty mesentery and *V. vulnificus* infection. Patients with misty mesentery caused by mesenteric panniculitis rarely exhibit acute abdominal symptoms.

**CPC-EM Capsule**

What do we already know about this clinical entity?

‘Misty-mesentery’ on abdominal computed tomography (CT) has a broad differential diagnosis, consisting of mesenteric panniculitis, neoplasms (mesenteric lymphoma, infiltration of lymphatics by gastrointestinal adenocarcinoma), mesenteric edema (secondary to portal hypertension), adjacent inflammation and idiopathic cause.

What makes this presentation of disease reportable?

Most etiologies of ‘misty-mesentery’ are relatively benign and non-life threatening. We are the first to propose such finding associated with an acute disease that carries grave-prognosis if treated inappropriately.

What is the major learning point?

‘Misty-mesentery’ on abdominal CT in a cirrhotic patient with unexplained abdominal pain may be an early clue to Vibrio vulnificus bacteremia, although the exact mechanism is still to be discovered.

How might this improve emergency medicine practice?

Recent sepsis guidelines reemphasize the importance of early administration of antbiotics in septic patients. Our proposed finding could help physicians select appropriate antibiotics, and potentially improve patient outcome.

Although both patients showed localized misty mesentery, their CT images and clinical conditions were non-suggestive of pancreatitis. Mesenteric edema due to liver cirrhosis was unlikely, because there was a lack of diffuse distribution of misty mesentery, subcutaneous edema and ascites.^8^ Previous CT also did not demonstrate any evidence of misty mesentery. Although the exact etiology and pathogenesis of the misty mesentery in our cases could not be identified, we suspect that the ingestion of uncooked seafood could have been the cause.

## CONCLUSION

We consider that our two patients’ intractable abdominal pain was related to misty mesentery caused by *V. vulnificus* infection. These cases emphasize the need to consider *V. vulnificus* bacteremia in cirrhotic patients with intractable abdominal pain, unexplained metabolic acidosis, and misty mesentery, so that appropriate antibiotics and aggressive resuscitation can be provided in a timely manner.
